# Perspectives on Physician-Assisted Suicide Among German Hospice Professionals: Findings from a Diagnostic Survey

**DOI:** 10.3390/healthcare13070763

**Published:** 2025-03-29

**Authors:** Janusz Surzykiewicz, Loren L. Toussaint, Elisabeth Riedl, Jean-Pierre Harder, Teresa Loichen, Arndt Büssing, Kathrin Maier, Sebastian Binyamin Skalski-Bednarz

**Affiliations:** 1 Faculty of Philosophy and Education, Catholic University of Eichstätt-Ingolstadt, 85072 Eichstätt, Germany; janusz.surzykiewicz@ku.de (J.S.); elisabeth.riedl@ku.de (E.R.); jean-pierre.harder@ku.de (J.-P.H.); teresa.loichen@ku.de (T.L.); sebastian.skalski@ku.de (S.B.S.-B.); 2 Faculty of Education, Cardinal Stefan Wyszyński University in Warsaw, 01938 Warsaw, Poland; 3 Department of Psychology, Luther College, Decorah, IA 52101, USA; 4 Professorship Quality of Life, Spirituality and Coping, Witten/Herdecke University, 58448 Herdecke, Germany; arndt.buessing@uni-wh.de; 5 Department of Educational Psychology in Social Work, Catholic University of Applied Sciences Munich, 80335 Munich, Germany; 6 Institute of Psychology, Humanitas University, 41200 Sosnowiec, Poland

**Keywords:** Physician-Assisted Suicide (PAS), hospice care, ethical challenges, attitudes toward PAS, latent class analysis

## Abstract

**Background/Objectives:** This study examines the attitudes of German hospice professionals toward physician-assisted suicide (PAS), focusing on ethical conflicts, emotional challenges, and institutional readiness within an unregulated legal context. **Methods:** A 2023 survey of 558 hospice workers used 13 closed-ended items to assess perspectives on PAS. **Results:** Latent class analysis identified subgroups based on shared response patterns. Three latent classes were identified, reflecting how hospice professionals might respond to the implementation of PAS in Germany: (1) “Low Conflict, Supportive Attitude”, referring to professionals likely to accept and support PAS with minimal ethical or emotional resistance; (2) “Moderate Conflict, Balanced Perspective”, describing professionals cautiously open to discussion but harboring notable ethical reservations; and (3) “High Conflict, Low Support”, encompassing professionals anticipated to face significant opposition and emotional challenges. Additionally, organizational readiness was reported as low (51.4%), alongside widespread concerns about increased workload and mental strain. **Conclusions:** Hospice professionals exhibit varied attitudes toward PAS, underscoring the importance of proactive measures should policymakers move toward its implementation. Ethical training, emotional support, and robust institutional frameworks will be essential to equip professionals for the challenges ahead.

## 1. Introduction

Assisted suicide, often referred to as physician-assisted suicide (PAS), remains a deeply contested issue, raising complex ethical, legal, and social questions worldwide. In regions where PAS is legally sanctioned, such as the Netherlands, Belgium, Luxembourg, Austria, Canada, Australia, and several U.S. states, the practice is governed by strict procedural safeguards. These include stages such as initial consultations, thorough assessment of patient eligibility, prescription of life-ending medication, and post-suicide support for grieving families [1,2,3]. Healthcare providers, particularly physicians and nurses, play central roles throughout this process, offering guidance, administering medications, and providing follow-up care for bereaved relatives [4].

In Germany, the issue of assisted suicide has sparked heated debate, reflecting broader societal tensions over autonomy and ethical boundaries. Between 2015 and 2020, PAS was restricted under a law that criminalized professional involvement, but the Federal Constitutional Court overturned this ban in 2020, asserting that the right to a self-determined death is a constitutional principle [5]. While this decision affirmed personal autonomy, it created significant legal ambiguity. Euthanasia remains explicitly prohibited, and PAS, though no longer criminalized, is currently unregulated due to legislative gridlock (with multiple attempts to establish a regulatory framework failing to secure majority support in the Bundestag in 2023 [6]). This lack of a clear framework places strain on healthcare providers, especially in palliative and hospice care, as they navigate ethical uncertainties and conflicting professional guidelines [7,8]. For instance, despite reports of right-to-die organizations facilitating approximately 350 cases in 2021, the absence of systematic documentation complicates efforts to evaluate the broader implications of PAS [7]. These unresolved issues highlight the urgent need for regulatory clarity.

In addition to legal ambiguities, cultural and religious contexts shape public and professional attitudes toward PAS, often resulting in stark differences in acceptance. Religiosity plays a key role, with Muslim communities, for example, categorically rejecting PAS and euthanasia based on the sanctity of life and the belief in divine authority over life and death [9]. In contrast, secular populations view PAS as an expression of personal autonomy and self-determination [10]. While autonomy refers to an individual’s capacity to make informed and voluntary decisions free from external coercion, self-determination emphasizes the active implementation of those decisions, particularly in contexts where individuals seek to exert control over their own end-of-life choices. Germany, as a relatively secular nation, has seen growing public acceptance of PAS, though religious beliefs still influence certain segments of the population [5]. These cultural nuances further complicate the debate, emphasizing the need to contextualize policy discussions within each country’s societal framework.

Hofmann et al. [5] offered pioneering insights into the attitudes of the German public, revealing broad support for PAS in cases of terminal illness but significant divisions concerning its use for mental health disorders or individuals who are “tired of life”. Survivors of suicide loss exhibited more cautious attitudes compared to the general population but acknowledged that PAS, when properly implemented, could offer a less traumatic alternative to traditional suicide, enabling families to say meaningful goodbyes and reducing guilt associated with sudden loss. However, localized studies on healthcare providers, particularly those in hospice settings, are lacking, requiring reliance on international research for preliminary insights.

Findings from countries such as Canada, the United States, Australia, and Austria reveal that healthcare providers often face emotional strain, ethical dilemmas, and increased workload when engaging with PAS. In Canada, Antonacci et al. [11] conducted a survey showing that nearly 75% of healthcare professionals had received patient requests for PAS, with many citing inadequate organizational support, insufficient training, and a lack of resources to manage the emotional toll of their involvement. Lavoie et al. [12] further found that personal ethical stances and institutional policies significantly influenced Canadian physicians’ willingness to participate in PAS. In the United States, Ganzini et al. [13] reported that 59% of hospice nurses had encountered requests for assistance with suicide, often describing substantial ethical and emotional challenges. Wilson et al. [14] analyzed Australian nurses involved in PAS-related care and found that while some were willing to participate, many expressed moral ambivalence or strong opposition, citing ethical concerns and emotional distress. Similarly, in Austria, where PAS was legalized in 2022, hospice and palliative care providers—particularly those in Christian-affiliated institutions—demonstrated significant reluctance, citing religious and ethical principles centered on the protection of life and dignity [3], similar to the theological opposition to PAS in Muslim communities mentioned earlier [5]. Viewing PAS as incompatible with palliative care, they instead emphasized symptom management and emotional support to alleviate patient suffering, often interpreting requests as expressions of existential distress rather than genuine end-of-life choices [3,15,16]. A systematic review of palliative care professionals’ experiences in regions where assisted dying is legal further underscores these complexities, showing that professionals often struggle to define when hastened death constitutes suicide and highlighting the emotional burden associated with such decisions [17].

Moreover, ethical debates surrounding PAS often focus on concerns about the so-called “slippery slope”, wherein eligibility for assisted dying might expand beyond terminal illnesses. Gorsuch [18] argues that the distinction between withdrawing life-sustaining treatment and actively assisting in suicide is not only legally but also morally justified. Similarly, Kim et al. [19] highlight that in countries such as Belgium and the Netherlands, regulatory frameworks for PAS have gradually evolved, raising questions about oversight and patient protection. Furthermore, Orentlicher [20] notes that while limiting PAS to terminally ill patients serves as a legal safeguard, it does not always resolve the complex ethical dilemmas faced by healthcare providers.

### Current Study

To address the limited understanding of healthcare providers’ attitudes toward PAS in Germany, we conducted a diagnostic survey focusing on hospice workers. As professionals at the forefront of end-of-life care, hospice workers often engage in discussions with patients and families about ethically complex decisions, including PAS [13,21]. Although they are unlikely to be directly involved in PAS under Germany’s current unregulated framework, the ongoing debate and interest from policymakers in adopting frameworks similar to those recently enacted in neighboring Austria [3] underscore the importance of understanding their perspectives. The central question guiding this research was “What are the distinct attitudes, ethical conflicts, emotional responses, and organizational readiness of German hospice workers toward PAS?” To explore their responses, we plan to use latent class analysis (LCA), a method that groups participants based on shared patterns in their attitudes, allowing for the identification of distinct categories that reflect diverse professional concerns and ethical considerations. A diagnostic survey is a systematic quantitative research method designed to describe and measure current attitudes, perceptions, and preparedness among specific populations. Quantitative data from such surveys are valuable because they provide precise, statistically reliable insights into the prevalence and distribution of attitudes and concerns, enabling evidence-based policymaking and targeted institutional interventions [22].

## 2. Materials and Methods

### 2.1. Participants

The study included a total of 558 hospice workers from 32 centers located across various federal states in Germany, ensuring broad coverage of professional perspectives relevant to end-of-life care. Participants were drawn from hospice care settings such as inpatient hospices, home-based hospice services, palliative care units, and day hospices, with representation from facilities in Bavaria, North Rhine-Westphalia, Baden-Württemberg, Saxony, Lower Saxony, and Brandenburg, reflecting a diverse regional distribution. The sample was predominantly female (64.2%). The average age of participants was 46.3 years (SD = 9.7), ranging from 22 to 65 years. Regarding marital status, the majority of participants were married or in long-term partnerships (63.5%). Educational background varied, with most participants holding qualifications in nursing or healthcare-related fields (68.1%). Participants had an average of 12.7 years (*SD* = 6.4) of work experience in hospice care, ranging from less than a year to over 30 years. Religious affiliation was also assessed, with a significant proportion identifying as Christian (56.3%). In terms of exposure to assisted suicide discussions, 54.3% of participants reported that patients “rarely” approached them to discuss assisted suicide. Detailed characteristics of the participants are presented in Table 1.

### 2.2. Data Collection Procedure

The survey was developed specifically as a diagnostic tool for this study to evaluate hospice workers’ attitudes toward PAS. The questionnaire was designed following a comprehensive review of the existing literature on PAS and healthcare provider perspectives, ensuring the inclusion of key themes and dimensions (e.g., [11,13]). It was distributed online via professional hospice networks and organizational mailing lists across Germany. Participation was voluntary and anonymous, and informed consent was obtained from all respondents prior to survey completion. The survey was conducted using the Qualtrics platform, which required participants to acknowledge an informed consent statement before proceeding to the questionnaire. This ensured that all respondents were fully aware of the study’s purpose, procedures, and data protection measures in line with General Data Protection Regulation (GDPR) standards. To safeguard data privacy, responses were anonymized, securely stored on encrypted servers, and accessible only to authorized researchers. The structured questionnaire consisted of 13 closed-ended items addressing attitudes toward PAS, alongside additional sociodemographic questions. Notably, apart from employment in hospice care, no additional recruitment requirements were imposed on participants. Data collection spanned a six-month period in 2023. Further details regarding ethical approval and Institutional Review Board (IRB) compliance are provided in the Institutional Review Board Statement section of this article.

### 2.3. Survey Instrument

The survey consisted of 13 closed-ended items specifically designed to assess hospice workers’ attitudes, concerns, and perceived preparedness regarding assisted suicide (PAS). The items aimed to explore a range of dimensions, including the frequency of patient inquiries, alignment with personal values, professional readiness, and the anticipated impact of PAS on their workload, mental well-being, and organizational structures. Participants responded using a 5-point option scale, with response formats tailored to the content of each question (“strongly disagree” to “strongly agree”, “never” to “very often”, or “very low” to “very high”). Although the questionnaire was originally developed specifically for this study and was not tested in a pilot study, this practice is common in diagnostic surveys aimed primarily at exploring current attitudes rather than validating psychometric instruments (e.g., [23]).

The survey covered diverse areas such as participants’ willingness to accompany patients during PAS, the potential for increased conflicts with patients, and their ability to discuss PAS openly and without judgment. Additional items assessed self-reported knowledge of PAS procedures and whether hospice organizations had begun considering structural adaptations to accommodate PAS. For example, one item asked respondents how often they had been approached by patients to discuss PAS, while others explored the perceived compatibility of PAS with personal values or the likelihood of PAS increasing professional workload.

Given the exploratory nature of the survey and its focus on capturing a broad range of attitudes across different domains, factor analysis was not conducted. The survey was designed as an exploratory tool rather than a psychometrically validated scale, with the primary goal of identifying subgroups of hospice workers holding unique attitudes. The structured format of the questionnaire facilitated a comprehensive understanding of these perspectives while also ensuring consistency across respondents. A complete list of survey items is presented in Table 2.

### 2.4. Statistical Analysis

The analysis began with frequency calculations for all survey items to summarize the distribution of responses and provide an overview of key trends among participants. Following this, LCA was conducted to identify distinct subgroups of respondents based on shared patterns in their responses. LCA allows for the identification of unobserved (latent) classes within the population, grouping participants with similar response patterns. Model fit was evaluated using the Akaike Information Criterion (AIC), the Bayesian Information Criterion (BIC), the sample-size adjusted BIC (SABIC), the Consistent AIC (CAIC), and the AIC with a third penalty term (AIC3). Lower values for these fit indices indicate a better-fitting model. Additionally, Log-Likelihood, chi-square (χ^2^), and G-squared (G^2^) statistics were reported to assess overall model performance and fit to the data. Descriptive statistics for each identified class were calculated to further characterize the subgroups in terms of sociodemographic and attitudinal variables. Post hoc comparisons were conducted to examine differences between latent classes, using chi-square tests for categorical variables and one-way ANOVA for continuous variables. Statistical significance was determined at a threshold of *p* < 0.05, with Bonferroni corrections applied to control for Type I error due to multiple comparisons. All analyses were performed using R version 4.2.2 [24]. The poLCA package was employed for latent class modeling, while base R functions were used for descriptive and inferential statistics.

## 3. Results

When asked how frequently patients approach healthcare professionals to discuss assisted suicide, the majority (54.3%) reported that such interactions occur “rarely”, while only 6.5% indicated they happen “often”. Regarding personal alignment with assisted suicide, responses varied widely, with 28.9% of participants stating they “somewhat agree” and 19.1% indicating they “strongly agree”. However, a substantial portion of respondents (40.7%) “strongly disagreed” with the compatibility of assisted suicide with their faith, spirituality, or religion.

Concerns about the potential impacts of assisted suicide were also evident. For example, 39.2% of respondents “somewhat agreed” and 31.9% “strongly agreed” that assisted suicide could be prematurely viewed as a solution rather than a last resort, reflecting significant ethical reservations. Similarly, concerns about mental burden and workload were notable, with 34.1% agreeing that they might become mentally burdened and 32.6% expressing concerns about increased workload following the implementation of assisted suicide. The prospect of increased conflicts with patients was another point of concern, with 31.7% “somewhat agreeing” and 6.5% “strongly agreeing” that such conflicts could arise.

Finally, responses revealed a lack of institutional readiness to adapt to PAS. Preliminary considerations for adapting organizational structures to accommodate assisted suicide were low, with 51.4% “strongly disagreeing” and 26.7% “somewhat disagreeing”. Detailed results for responses to all survey items are presented in Table 2.

LCA was conducted to group participants into meaningful subgroups based on shared patterns in their responses. The results, summarized in Table 3, identified three latent classes as the optimal solution. This determination was supported by the lowest values for AIC, AIC3, and CAIC, alongside favorable BIC and SABIC values, which collectively indicated better model parsimony. Log-Likelihood values supported this conclusion by showing improved fit with the addition of three classes. Although G^2^ and χ^2^ values were significant, which may suggest some deviations between the model and the observed data, these measures are highly sensitive to sample size. Consequently, they are less reliable for deciding the best-fitting model in larger datasets and are often interpreted alongside penalized criteria such as AIC and BIC [25]. Therefore, the three-class solution was selected as it offered the best balance between model simplicity and fit, enabling the classification of distinct attitudes and concerns among participants regarding PAS.

**Figure 1:**
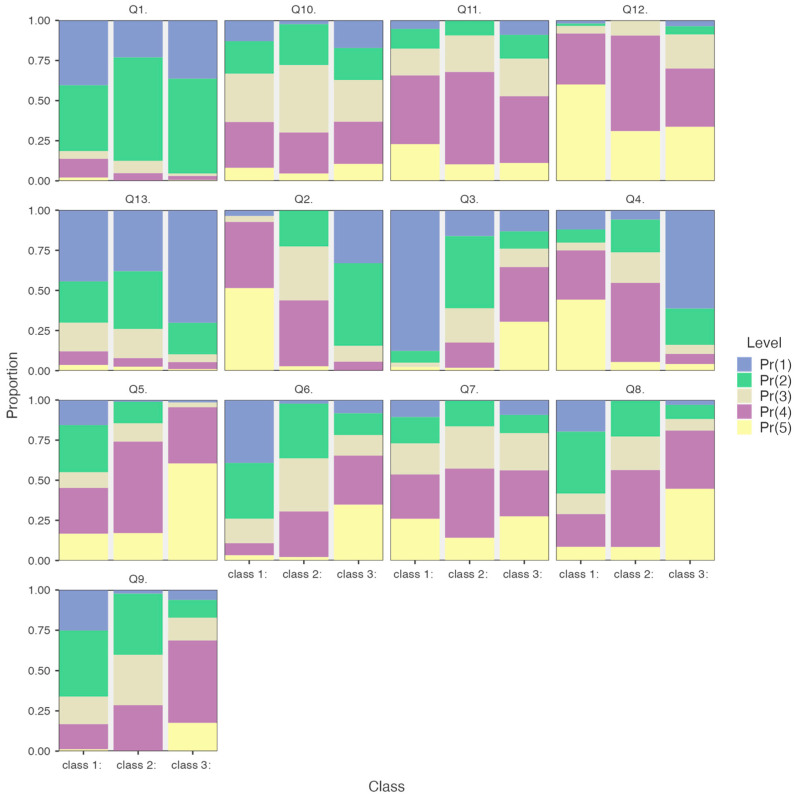
Comparison of mean responses for each survey item across latent classes.

The post hoc analyses reported in Table 4 revealed notable differences between the three latent classes, highlighting distinct attitudes and concerns regarding assisted suicide. Class 1 (“Low Conflict, Supportive Attitude”) displayed confidence and readiness to support patients, demonstrating lower levels of emotional and ethical conflict compared to other groups. Class 2 (“Moderate Conflict, Balanced Perspective”) represented a middle ground, combining openness to discussion with ethical reservations, reflecting a cautious but balanced approach to addressing assisted suicide in professional settings. In contrast, class 3 (“High Conflict, Low Support”) showed significant discomfort with assisted suicide, characterized by heightened concerns over emotional strain and a perceived misalignment with personal and professional values, reflecting strong opposition to the practice. These distinctions underscore the diverse perspectives and challenges faced by hospice professionals, emphasizing the need for tailored strategies to support each group effectively. Figure 1 graphically compares the mean responses for each survey item across latent classes, illustrating the distinct response patterns observed.

## 4. Discussion

This study examined the attitudes of German hospice professionals toward PAS, revealing diverse perspectives shaped by personal, ethical, and professional factors. The findings point to significant ethical and emotional challenges, possibly exacerbated by the absence of a unified legal framework governing PAS in Germany. Hofmann et al. [5] highlight the public’s generally cautious yet supportive stance toward PAS when strict safeguards are in place, but the lack of institutional preparedness adds an additional layer of uncertainty for professionals tasked with managing these complex issues.

A key finding of this study revealed that over half of the participants strongly disagreed that their hospice organizations had taken steps toward considering adaptations for PAS, pointing to a perceived lack of readiness to address the ethical and procedural challenges that potential PAS regulation in Germany might bring. In contrast, Austria has already established a legal framework for assisted dying, which has compelled healthcare providers to address these challenges in practice. Studies from Austria reveal that the introduction of assisted dying legislation brought to light significant gaps in ethical guidance and professional preparedness, initially leading to uncertainty and tension among healthcare workers [15,16]. These challenges underscored the importance of consultation services, training programs, and the development of institutional policies to support professionals managing PAS-related cases. Germany, without an established PAS framework, has the opportunity to preemptively address such challenges by implementing clear ethical guidelines and comprehensive preparatory measures to ensure healthcare providers are supported in the event of future legislation.

Another critical dimension revealed by the study pertains to the psychological impact of PAS on hospice professionals. Over one-third of participants expressed apprehension about the emotional burden that engagement with PAS might entail. This finding aligns with research from Canada, which highlights that professionals working in environments lacking sufficient training and organizational resources often face elevated stress levels when dealing with ethically fraught issues like PAS [11]. The Canadian experience suggests that targeted interventions, such as communication-focused training and the establishment of explicit protocols, can alleviate these pressures and foster a more resilient workforce capable of navigating ethically challenging scenarios.

The potential for conflicts between hospice professionals, patients, and families also emerged as a significant concern, particularly when differing expectations and values around PAS arise. Evidence from the Netherlands and Austria similarly highlights how PAS can conflict with the core philosophy of palliative care, which emphasizes the alleviation of suffering without actively hastening death [3,4]. Addressing this discord requires clear ethical guidelines and effective communication strategies to help professionals navigate conflicting priorities while maintaining trust and compassion in patient care.

A closer analysis of the identified profiles of attitudes toward PAS among hospice workers provides valuable insights into how these challenges might be effectively addressed. The “Low Conflict, Supportive Attitude” group emerged as the most practically positioned to engage constructively with PAS, should policymakers move toward its implementation. This group displayed relatively low levels of ethical conflict and emotional strain, indicating their capacity to approach PAS discussions in a thoughtful and balanced manner. In contrast, the “High Conflict, Low Support” group exhibited significant ethical and emotional challenges, emphasizing the need for tailored interventions, such as emotional counseling, peer support, and access to ethical consultation services. Meanwhile, the “Moderate Conflict, Balanced Perspective” group reflected a mix of cautiousness and openness, suggesting that additional resources and structured guidance could further strengthen their ability to contribute effectively to PAS-related discussions. Interestingly, despite the marked differences in ethical concerns and levels of emotional strain across the groups, their knowledge about PAS did not differ significantly. This suggests that while education is essential to provide all professionals with procedural understanding, addressing ethical dilemmas and fostering patient-centered perspectives may play a crucial role in helping hospice workers navigate PAS-related issues with confidence.

These findings correspond with Wilson et al.’s [14] study on Australian registered nurses’ responses to assisted dying requests, which identified five professional profiles: Facilitator, Complier, Expediter, Objector, and Detached. Their study, conducted on more than 350 experienced nurses primarily working in end-of-life, tertiary, and community care settings, similarly found that ethical concerns, emotional strain, and professional obligations influenced how nurses responded to PAS. Both studies demonstrate that healthcare professionals do not form a homogeneous group in their approach to PAS but rather cluster into distinct profiles that reflect varying levels of ethical conflict, emotional burden, and willingness to engage in PAS-related discussions. More specifically, the “Low Conflict, Supportive Attitude” group in the present study aligns with Wilson et al.’s [14] “Facilitator” profile, which also exhibited low ethical conflict and a greater readiness to engage in PAS discussions. Conversely, the “High Conflict, Low Support” group mirrors their “Objector” profile, characterized by strong moral opposition and reluctance to participate in PAS-related decisions. The “Moderate Conflict, Balanced Perspective” group corresponds to Wilson et al.’s [14] “Complier” profile, suggesting that structured guidelines and institutional support could help these professionals navigate PAS dilemmas more effectively.

An important consideration in this study is the deliberate exclusion of sociodemographic factors from the LCA. This approach aimed to prevent these variables from overshadowing the exploration of ethical and emotional concerns, particularly as the survey directly addressed alignment with personal values and religious beliefs. While this strategy preserved the focus on core concerns, it underscores the value of future research integrating sociodemographic contexts to enrich our understanding of how such factors shape attitudes toward PAS.

Regarding limitations, a notable issue is the lack of control for specific professional roles within hospice care, which could influence the intensity of concerns and their prioritization. Future studies should consider accounting for these roles, as it is reasonable to assume that physicians and trained healthcare professionals are more prepared to focus on patient needs, whereas volunteers—who constitute the majority of hospice staff in Germany—may respond to PAS scenarios based more on personal beliefs. According to available data, the number of volunteers involved in hospice work in Germany is estimated to exceed 100,000 individuals, whereas the number of physicians specialized in palliative care is significantly smaller, with approximately 9000 practitioners across the country [26,27]. For further insights into the role of volunteers in German inpatient hospice and palliative care, see [28]. Although volunteers would not typically be directly involved in PAS, their roles in patient discussions and supporting grieving families are critical and warrant further exploration. Additionally, the quantitative data provide a systematic and structured assessment of the prevalence and intensity of ethical concerns related to PAS among hospice workers; however, they do not fully capture the specific ethical dilemmas or the contextual complexities in which these concerns arise. Since our study focused on measuring the overall intensity of ethical conflicts rather than identifying their precise nature, future research could integrate both quantitative and qualitative approaches to explore the concrete ethical challenges hospice professionals encounter in PAS-related scenarios, offering a deeper understanding of their decision-making processes and moral reasoning. Another limitation involves potential circularity in the LCA, as the same indicators used to form the classes were also tested for differences across them. This could lead to apparent differences that are less informative; however, exploring the most divergent indicators still provides valuable insights into the unique characteristics of each class [29].

## 5. Conclusions

The findings of this study provide valuable insights into the diverse attitudes of hospice professionals toward physician-assisted suicide (PAS), revealing significant ethical and emotional complexities. However, these results should be interpreted with caution as they reflect the perspectives of a specific sample within the German hospice care context, a country where PAS has not yet been officially implemented. While the identification of distinct latent classes offers a useful framework for understanding variations in professional attitudes, further research is needed to explore how these differences might influence practical decision-making and patient care if PAS were to be introduced.

The findings indicate that hospice professionals’ attitudes toward PAS are diverse but can be systematically categorized into three latent profiles, each reflecting distinct ethical concerns, emotional burdens, and institutional readiness. These variations underscore the need for tailored interventions to support different professional groups in navigating the complexities of PAS within hospice care. Furthermore, the consistency in knowledge levels across groups suggests that while education about PAS is indeed important, addressing professionals’ ethical dilemmas and emotional challenges may be key to helping them navigate this sensitive issue effectively.

## Figures and Tables

**Table 1:** Demographic characteristics of participants.

Characteristic	Percentage (%) or Mean (*SD*)
Gender	
Female	64.2
Male	35.8
Age	
Years	46.3 (9.7)
Marital Status	
Married/Partnership	63.5
Single	24.8
Divorced/Widowed	11.7
Educational Background	
Nursing/Healthcare	68.1
Advanced Degrees	18.4
Other Training	13.5
Work Experience (years)	12.7 (6.4)
Religious Affiliation	
Christian	56.3
No Affiliation	32.9
Other Religions/Spiritual	10.8
Exposure to Assisted Suicide Discussions	
Rarely	54.3
Occasionally	31.2
Never	8
Often	6.5

**Table 2:** Distribution of responses for assisted suicide-related questions (percentages).

**Question**	**Never** **(1)**	**Rarely** **(2)**	**Neutral (3)**	**Often** **(4)**	**Very Often (5)**
1. How often have patients approached you to discuss assisted suicide?	33.9	54.3	4.5	6.5	0.8
**Question**	**Strongly disagree (1)**	**Somewhat disagree ** **(2)**	**Neutral (3)**	**Somewhat agree ** **(4)**	**Strongly agree ** **(5)**
2. Assisted suicide aligns with my personal values.	12.7	24.6	14.7	28.9	19.1
3. Assisted suicide is not compatible with my faith, spirituality, or religion.	40.7	19.7	11.3	16.5	11.8
4. I would accompany a patient during the process of assisted suicide.	27.2	16.8	9.3	27.8	18.9
5. Assisted suicide can be prematurely viewed as a potential solution rather than the absolute last resort in a hopeless situation.	6.5	14.5	7.9	39.2	31.9
6. Patients’ and relatives’ trust in the quality of care may decrease after the implementation of assisted suicide.	17.6	27.2	19.7	21.7	13.8
7. Assisted suicide may lead to new demanding tasks that will further increase my workload.	7.2	14.5	22.8	32.6	22.9
8. In the new work situation shaped by assisted suicide, I may quickly become mentally burdened.	8.2	23.5	13.3	34.1	20.9
9. Assisted suicide may lead to increased conflicts with patients.	11.8	29.7	20.3	31.7	6.5
**Question**	**Very low (1)**	**Rather low (2)**	**Neutral (3)**	**Rather high (4)**	**Very high (5)**
10. How would you assess your knowledge of the procedures and the resulting requirements for hospice work about assisted suicide?	11.3	21.7	32.3	26.9	7.8
**Question**	**Strongly disagree (1)**	**Somewhat disagree ** **(2)**	**Neutral (3)**	**Somewhat agree ** **(4)**	**Strongly agree ** **(5)**
11. I can recognize and appropriately address the needs of patients considering assisted suicide.	5	12.4	20.8	46	15.8
12. I can discuss a patient’s desire for assisted suicide openly and without judgment.	2	2.3	11.8	41.6	42.3
13. There are preliminary considerations for adapting organizational structures in the hospice where I work regarding the implementation of assisted suicide.	51.4	26.7	13.4	6.2	2.3

**Table 3:** Fit indices for latent class analysis (LCA) models.

Class	AIC	AIC3	BIC	SABIC	CAIC	Log-Likelihood	Χ^2^	G^2^
1	20,591	20,643	20,815	20,650	20,867	–10,243	7.19	13,434
2	19,717	19,824	20,180	19,840	20,287	–9752	2.39	12,452
3	19,039	19,201	20,040	19,326	20,202	–9308	1.18	11,570
4	19,370	19,587	20,309	19,620	20,526	–9468	1.65	11,951
5	19,148	19,420	20,324	19,461	20,596	–9502	1.09	11,987

**Table 4:** Means and significant differences between latent classes for each item (post hoc pairwise comparisons).

Question	A: Class 1 (*M* ± *SD*); *n* = 198	B: Class 2 (*M* ± *SD*); *n* = 164	C: Class 3 (*M* ± *SD*); *n* = 196	*F*_(2,359)_	Post-Hoc
1.	1.93 (1.05)	1.95 (0.71)	1.72 (0.67)	5.42 **	A > C *, B > C *
2.	4.4 (0.85)	3.23 (0.8)	1.88 (0.8)	461.65 ***	A > B ***, A > C ***, B > C ***
3.	1.2 (0.67)	2.43 (0.98)	3.57 (1.37)	273.92 ***	A < B ***, A < C ***, B < C ***
4.	3.88 (1.37)	3.32 (0.99)	1.68 (1.1)	184.63 ***	A > B ***, A > C ***, B > C ***
5.	2.99 (1.36)	3.76 (0.93)	4.53 (0.7)	113.39 ***	A < B ***, A < C ***, B < C ***
6.	1.98 (1.04)	2.93 (0.9)	3.71 (1.28)	112.86 ***	A < B ***, A < C ***, B < C ***
7.	3.44 (1.32)	3.54 (0.93)	3.52 (1.27)	0.35	--
8.	2.59 (1.24)	3.39 (0.94)	4.12 (1.07)	86.31 ***	A < B ***, A < C ***, B < C ***
9.	2.26 (1.04)	2.84 (0.85)	3.63 (1.08)	82.91 ***	A < B ***, A < C ***, B < C ***
10.	2.98 (1.16)	3.02 (0.91)	2.95 (1.24)	0.23	--
11.	3.65 (1.12)	3.68 (0.8)	3.33 (1.12)	6.59 **	A > C **, B > C **
12.	4.45 (0.83)	4.23 (0.6)	3.92 (1.04)	15.48 ***	A > B *, A > C ***, B > C **
13.	2.01 (1.14)	1.99 (0.99)	1.46 (0.86)	21.31 ***	A > C ***, B > C ***

Note. * *p* < 0.05, ** *p* < 0.01, *** *p* < 0.001.

## Data Availability

The data supporting the findings of this study are available upon reasonable request from the author, J.S. Due to privacy and ethical restrictions, the dataset is not publicly available.

## Abbreviations

The following abbreviations are used in this manuscript:

PAS	Physician-Assisted Suicide
LCA	Latent Class Analysis
AIC	Akaike Information Criterion
CAIC	Consistent Akaike Information Criterion
BIC	Bayesian Information Criterion
SABIC	Sample-Size Adjusted BIC
AIC3	AIC With a Third Penalty Term
*χ*^2^	Chi-Square
*G*^2^	G-Squared
